# Skin Pigmentation Types, Causes and Treatment—A Review

**DOI:** 10.3390/molecules28124839

**Published:** 2023-06-18

**Authors:** Amin Mahmood Thawabteh, Alaa Jibreen, Donia Karaman, Alà Thawabteh, Rafik Karaman

**Affiliations:** 1 Faculty of Pharmacy, Nursing and Health Professions, Birzeit University, Ramallah 00972, Palestine; athawabtah@birzeit.edu; 2 General Safety Section, General Services Department, Birzeit University, Bir Zeit 71939, Palestine; 3 Research and Development Department, Beit Jala Pharmaceutical Co., Ltd., Beit Jala 97300, Palestine; amfareh@beitjalapharma.com; 4 Pharmaceutical Sciences Department, Faculty of Pharmacy, Al-Quds University, Jerusalem 20002, Palestine; kdonia65@yahoo.com; 5 Medical Imaging Department, Faculty of Health Profession, Al-Quds University, Jerusalem 20002, Palestine; athawabteh@staff.alquds.edu; 6 Department of Sciences, University of Basilicata, Via dell’Ateneo Lucano 10, 85100 Potenza, Italy

**Keywords:** skin pigmentation, melanin, tyrosinase inhibitors, hypopigmentation, hyperpigmentation, vitiligo, skin lightening, depigmentation

## Abstract

Human skin pigmentation and melanin synthesis are incredibly variable, and are impacted by genetics, UV exposure, and some drugs. Patients’ physical appearance, psychological health, and social functioning are all impacted by a sizable number of skin conditions that cause pigmentary abnormalities. Hyperpigmentation, where pigment appears to overflow, and hypopigmentation, where pigment is reduced, are the two major classifications of skin pigmentation. Albinism, melasma, vitiligo, Addison’s disease, and post-inflammatory hyperpigmentation, which can be brought on by eczema, acne vulgaris, and drug interactions, are the most common skin pigmentation disorders in clinical practice. Anti-inflammatory medications, antioxidants, and medications that inhibit tyrosinase, which prevents the production of melanin, are all possible treatments for pigmentation problems. Skin pigmentation can be treated orally and topically with medications, herbal remedies, and cosmetic products, but a doctor should always be consulted before beginning any new medicine or treatment plan. This review article explores the numerous types of pigmentation problems, their causes, and treatments, as well as the 25 plants, 4 marine species, and 17 topical and oral medications now on the market that have been clinically tested to treat skin diseases.

## 1. Introduction

Skin pigmentation, which refers to how much melanin the body generates, determines the color of the skin. The two main types of melanin, eumelanin, and pheomelanin, are produced by melanocytes in the epidermal layer of the skin. Pheomelanin causes lighter skin tones, while eumelanin is responsible for darker skin tones [1,2].

**Figure 1:**
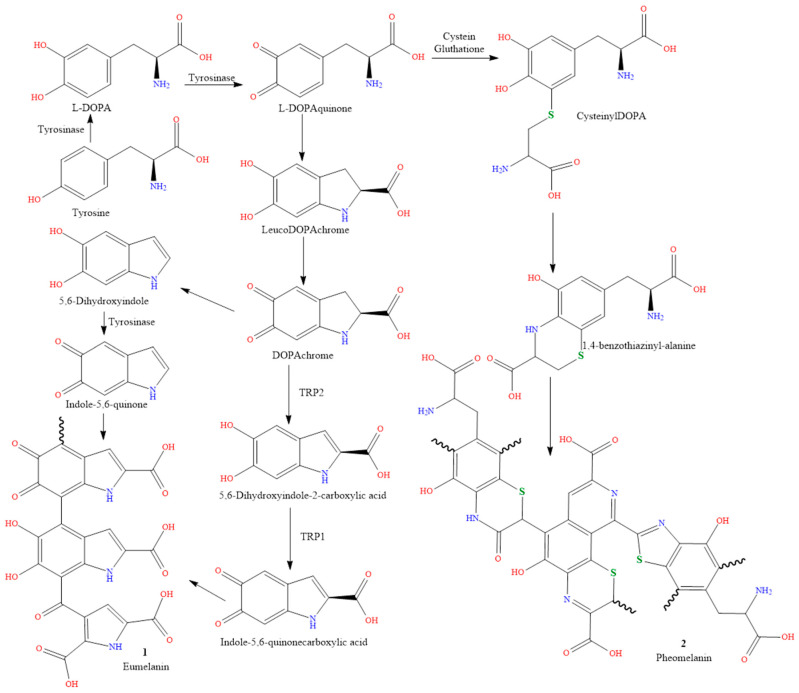
Schematic representation of the melanin synthesis pathway.

The skin is protected from sunburn by the dark brown pigment eumelanin (compound **1** in Figure 1), which absorbs UV rays from the sun. Darker skin tones are related to higher levels of eumelanin, while lighter skin tones are associated with lower levels. The capacity of eumelanin to prevent skin cancer is one of its additional benefits. Studies have shown that those with higher levels of eumelanin had a lower chance of developing skin cancer than people with lower levels. By absorbing solar heat, and keeping the body cool, eumelanin also helps to regulate body temperature [3,4].

The pigment pheomelanin has a lighter yellow-red tint (compound **2** in Figure 1). Because pheomelanin does not absorb UV rays as effectively as eumelanin, those with higher levels have lighter skin tones, and are more prone to skin damage and sunburn. Pheomelanin does, however, have certain benefits. It helps to control body temperature, and can keep the body cool in hot conditions by reflecting heat away from the body. Pheomelanin can also help prevent melanoma and other types of skin cancer [5,6].

The gene that controls melanin production for the melanocortin G-protein-coupled receptor 1 (MC1R) is located at gene locus q24.3 on chromosome 16. The MC1R gene regulates tanning (sensitivity to light exposure and sunburn), governs skin and hair color, and raises the risk of melanoma [7,8].

The rates of melanin synthesis vary across members of the same family, and between racial groups (Figure 1). This variation (MSH) is caused by genetics, sun exposure, and certain hormones that stimulate melanocytes, such as adrenocorticotropic hormone (ACTH), lipotropin, and melanocyte-stimulating hormone. Using more melatonin results in a grayish-brown skin tone [8,9,10].

## 2. Causes of Skin Pigmentation

Skin pigmentation is a common condition that can be triggered by various factors. The three leading causes of skin pigmentation are genetics, sun exposure, and particular medications. Understanding the fundamental causes of skin pigmentation will help us understand how to treat and prevent it [2].

### 2.1. Genetics

Unexpectedly, 125 genes can influence skin tone. The production of melanin, as depicted in Figure 1, is governed by genes and hormones. A person has control over his or her skin’s ability to function and live, as well as how much pheomelanin or eumelanin they produce by, for instance, deciding how much sun exposure they receive, or the amount of drugs and cosmetics they use. These elements could alter the tone of skin over time [1]. Thus, one of the most frequent reasons for skin color is genetics. Genetics may be able to predict how many melanocytes each individual will have. It is melanocytes, which are skin cells, that make melanin. However, during hyperpigmentation and tanning, melanosomes (the organelles that contain melanin) must be transported and expanded, but during hypopigmentation, melanosomes decrease [11]. Melanin, the pigment that gives skin its color, is more likely to be present in larger concentrations in people with darker skin tones. For example, individuals with darker skin tones frequently have higher melanin levels than those with lighter skin tones [12,13,14].

### 2.2. Sun Exposure

**Figure 2:**
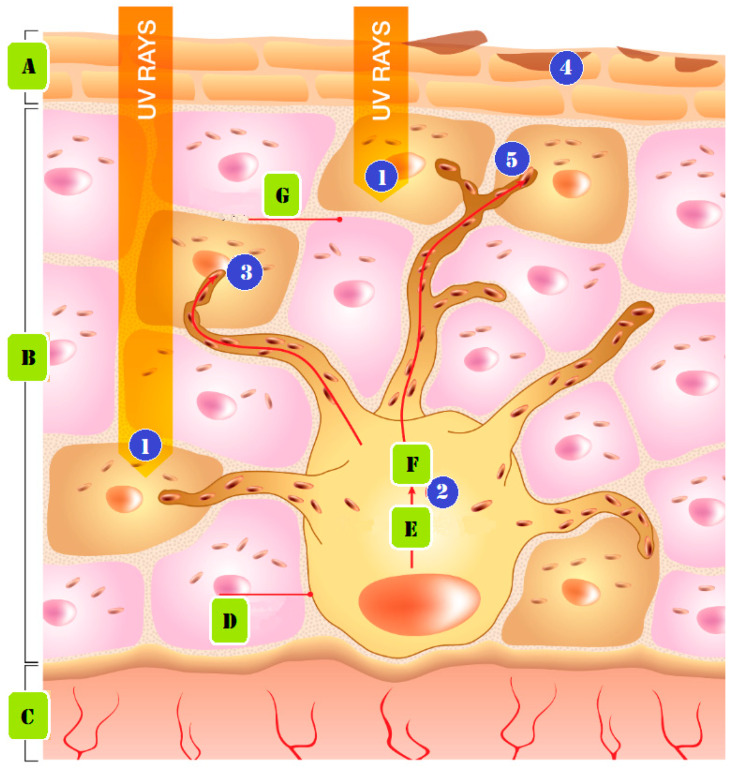
Pigmentation from prolonged UV exposure formed: (A) stratum corneum, (B) epidermis, (C) dermis, (D) melanocyte cell, (E) tyrosinase and tyrosine, (F) melanin, and (G) keratinocyte cell.

Sun exposure is a common cause of skin pigmentation. The body produces more melanin, in order to defend itself against UV rays from the sun. This may make the skin more pigmented, to shield it from the sun’s rays. Figure 2 demonstrates how persistent UV exposure leads to the emergence of pigmentation. The following phases make up the formation mechanism. (1) UV radiation produces free radicals. (2) The free radicals and UV light activate biological agents that impact melanocytes, the cells responsible for creating pigment. (3) The enzyme tyrosinase transforms the amino acid tyrosine into melanin pigments, which can be either red or brown in color. (4) Biological substances act to increase the activity of the enzyme Tyrosinase, which generates pigment. (5) Melanin is lost from the skin, as skin cells travel to the surface layers and are shed during the skin’s natural exfoliation process. Melanin is delivered as granules from nearby keratinocytes, to give the skin its color [4,14,15].

### 2.3. Medications

Several medications may also lighten the skin’s pigment. One class of drugs, antibiotics, can boost melanin synthesis, increasing skin color. When certain medications, such as birth control pills, are taken together, skin pigmentation may also intensify. A person taking medicine should speak with their doctor to find out if the medication could impact the color of their skin [16,17].

## 3. Types of Pigmentation Disorders

**Figure 3:**
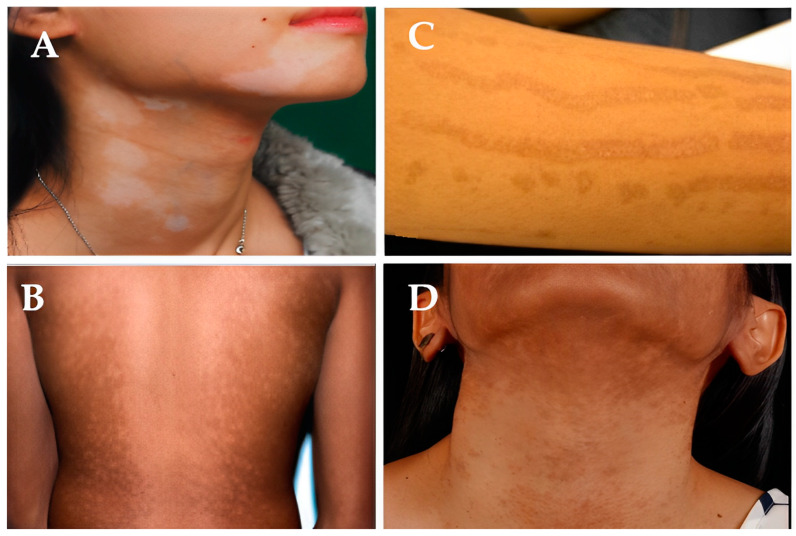
Hypopigmentation cases (**A** [22], **B** [23]) and hyperpigmentation cases (**C**,**D** [24]). (**A**) Reproduced with permission from Joo-Heung Lee, Cho-Rok Kim, Dong-Youn Lee, Photodermatology, Photoimmunology & Photomedicine; published by John Wiley and Sons, 2011. (**B**) Reproduced with permission from Jeremy P Hill, Jonathan M Batchelor, The BMJ; published by BMJ Publishing Group Ltd., 2017. (**C**,**D**) Reproduced with permission from Narumol Silpa-archa, Indermeet Kohli, Suteeraporn Chaowattanapanit, Henry W. Lim, Iltefat Hamzavi, Journal of the American Academy of Dermatology; published by Elsevier, 2017.

While they are ill, a person’s skin tone may alter, becoming lighter (hypopigmentation), as seen in Figure 3A,B, or darker (hyperpigmentation), as seen in Figure 3C,D. Melanin, the pigment that regulates skin color, is produced less frequently by the body, which results in hypopigmentation. Hyperpigmentation, on the other hand, is an increase in melanin synthesis [4,5,18].

### 3.1. Causes of Hypopigmentation

**Figure 4:**
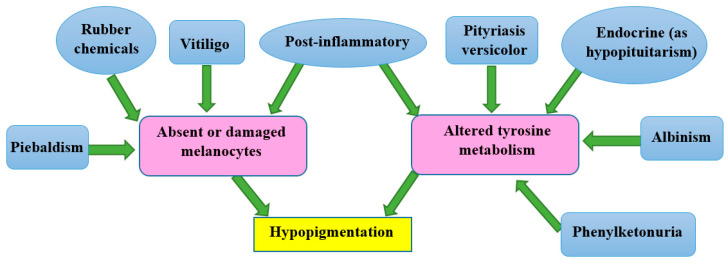
The mechanisms involved in some types of hypopigmentation.

Prior skin trauma, including skin sores such as blisters, infections, burns, exposure to chemicals, and other wounds, is the most common cause of low melanin content (hypopigmentation). After an injury has healed, the skin will be paler than the surrounding skin surface [19]. Other genetic diseases can result in hypopigmentation in different parts of the skin. As seen in Figure 3, hereditary disorders such as albinism, melasma, fungal infections, pityriasis versicolor, pityriasis alba, and vitiligo can result in hypopigmentation, as seen in the mechanism in Figure 4. At birth, albinism is caused by a genetic abnormality known as low melanin concentration. The prevalent physical traits of albinos include a white complexion, dark blue eyes, and white hair [20,21]. The genetic melasma condition can cause brown or blue-gray spots to develop on a person’s arms or face. Hormones, sun exposure, or contraceptive medication may bring it on [22,23].

Despite the fact that the Malassezia genus is responsible for the widespread fungal infection known as tinea versicolor, also known as pityriasis versicolor, it is possible for fungi to infect humans, and change the color of their skin. Small regions of discoloration are brought on by Malassezia’s alteration of the skin’s normal melanin pigmentation. The patches on the shoulders and buttocks may be lighter or darker than the overall healthy skin tone [25]. Pityriasis alba is a skin condition that typically affects adolescents and teenagers, and is characterized by oval or circular hypopigmented lesions with soft scales. Lesions on the face, upper body, and arms, which are more noticeable in those with darker skin tones, may be modestly erythematous, before becoming hypopigmented [26].

**Figure 5:**
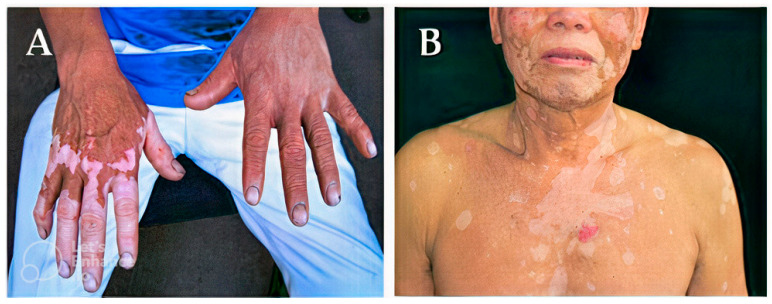
Depigmentation cases [27,28]. (**A**) Reproduced with permission from Louise McMichael, the BMJ; published by BMJ Publishing Group Ltd., 2012. (**B**) Reproduced with permission from Jing Jing, Xiao-Yong Man, the BMJ; published by BMJ Publishing Group Ltd., 2021.

Depigmentation, which happens when the skin entirely loses pigment and turns white, is another prevalent hypopigmented skin disease. An example of this is the auto-immune condition vitiligo, which is characterized by macules of a white chalky substance on the skin, and melanocyte loss, a common cause of depigmentation. Vitiligo causes smooth, white patches to appear on the skin, as illustrated in examples A and B in Figure 5. Many times, vitiligo is dismissed as a minor problem [27,28].

### 3.2. Causes of Hyperpigmentation

A rise in melanin production causes hyperpigmentation. Examples C and D in Figure 3, and other situations where melanin synthesis increases, primarily result from sun exposure, dermatological conditions, hormones, age, hereditary factors, skin injuries or inflammation, and acne [24]. The most frequent cause of hyperpigmentation is exposure to the sun, which heavily stimulates melanin production. A recent study (Figure 2) demonstrated how early sun exposure might worsen dark spots, by making them resemble melasma, post-inflammatory spots, and age spots [24]. Two examples of hyperpigmentation brought on by hormonal factors are chloasma and melasma. It has been established that the female sex hormones estrogen and progesterone, which boost melanin formation when the body is exposed to sunshine, are to blame for this disease, which is prevalent in women. Hormone replacement therapy has the adverse effect of hyperpigmentation [29].

Melanocyte counts decline with age, but those that are still present grow and specialize. These physiological alterations show how aging becomes increasingly obvious in people over 40 [30]. Genetics has an impact on pigmentation. The development of the melanocyte function, which impacts skin color, requires particular genes [31]. According to the term “post-inflammatory hyperpigmentation”, this appears following several types of skin inflammation or injury, including chemical exposure, burns, wounds, psoriasis or atopic dermatitis, and acne. The skin looks more discolored and blacker after the lesion has healed [28]. The innermost layer of the skin, the dermis, can get infected by papules, pustules, and acne. Unusually dark spots form when skin diseases induce an abnormally high melanin synthesis. Similarly to this, the real causes of the hyper-pigmentation problem are infections of the fatty glands and hair follicles. Mild acne typically doesn’t cause hyperpigmentation. Acne lesions that have been squeezed, squashed, or penetrated will also darken and become more pigmented [32]. Some causes of hyperpigmentation include pregnancy-related birthmarks, age spots, acne scars, and a number of drugs, including antibiotics, birth control pills, antimalarials, and tricyclic antidepressants. A rare condition called Addison’s disease results in black skin patches and decreased adrenal gland activity. Hyperpigmentation can occasionally occur after laser or light treatment [33].

## 4. Drugs for Treatment of Skin Pigmentation

Despite being well-recognized for many years, drugs for skin pigmentation have only recently become more widely available. Topical creams and oral pills are the primary medications for skin pigmentation. It would be best to balance the advantages and disadvantages of both medicines to choose which is most beneficial [33,34].

### 4.1. Oral Medications

Oral medicines are a potential substitute for treating skin ailments, and modifying skin tone. Such drugs are beneficial because they are more potent than topical creams, and do not need to be applied or disposed of as frequently as topical creams. However, there are certain drawbacks to taking oral medications. They can be expensive and cause more significant side effects than topical therapies [35].

**Figure 6:**
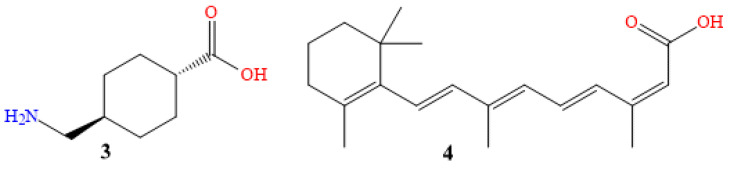
Chemical structures of tranexamic acid (**3**), and isotretinoin (**4**).

Compound **3** in Figure 6 is tranexamic acid (Traxamac^®^ 250 mg), one of several coagulation modifiers. In addition to its use on eczema, melasma, other associated ailments, toxic reactions, and urticaria, and its effects on erythema, itching, swelling, and other recognized symptoms, it has also been used to treat various illnesses. A plasmin inhibitor, tranexamic acid prevents the plasminogen activator from converting plasminogen to plasmin, by reversibly shutting off lysine binding sites on plasminogen molecules. This reduces atypical fibrinolysis, and prevents blood loss. According to recent studies, tranexamic acid helps tyrosinase to untangle tangles. It might avoid and stop hyperpigmentation, by reducing melanin production. It is a widely used pharmaceutical technique that is easily accessible and effective against pigment spots. Although it inhibits the effects of tyrosinase, and changes the relationship between keratinocytes and melanocytes, it decreases dermal vascularity, and lessens melanin production [36,37,38,39,40].

Using tranexamic acid orally, in a dosage of 250 mg twice daily for six months, on 75 patients, a clinical and photographic evaluation revealed an initial decline in melasma after the first month for 82.4% of patients, and 94.6% in the second month. The development of pigmentation had been used to measure the treatment’s success (excellent if >90%, good if >60%, fair if >30%, and poor if 30%). After six months, the overall development rate was 95.9%, with 10.8% being excellent, 54% being good, and 31.1% being fair, which is evidence that oral tranexamic acid is a safe and effective melasma treatment [41,42].

Mexameter^®^ was utilized to evaluate the suggested lesional melanin index (MI) ranks, and the erythema index (EI) scores, for 25 patients who received 1500 mg twice daily for two months. Both of these scores fell off dramatically. Histological examination confirmed significant decreases in mast cell counts, vessels, and epidermal pigmentation. Here is an illustration of how oral tranexamic acid reversed melasma-related dermal changes, including increased vascularity, decreased mast cell populations, and decreased melasma-related epidermal pigmentation [43,44].

In this study of 25 women, certain sides of the face received twice-daily applications of 5% topical tranexamic acid for 12 weeks, as a melasma treatment. The Mexameter and Melasma Area and Severity Index (MASI) results revealed a notable drop in the MI and MASI scores. Additionally, for 12 weeks, 23 melasma patients applied a 2% tranexamic acid emulsion twice daily to their whole faces. With a rise in the lightness values, and a decrease in the erythema values, the mMASI and chromameter results showed a significant improvement in the fourth and eighth weeks [45,46,47].

Isotretinoin is the 13-cis-retinoic acid derivative of vitamin A (Isotane^®^ 20 mg, molecule **4** in Figure 6) [48,49,50]. In treating acne vulgaris, oral isotretinoin exerts its effects by reducing sebaceous gland activity, the development of *Propionibacterium acnes*, and inflammation. This facilitates pore cleaning, and inhibits the growth of new lesions [51,52,53].

The administration of 20 mg of Accutane (isotretinoin) orally was randomly assigned to 60 patients (aged 35 to 65); 42 of the women, and 18 of the men. It was administered three times a week for no more than two months, and tracking continued for months after the study was over. The 60 patients reported reductions in their wrinkles, pore thickness, and pore size. They noticed that the skin became significantly smoother and lighter in color. Both the elasticity and the tone of the skin improved. Additionally, they noticed a decrease in pigmented lesions and hyperpigmentation [54,55].

The severity of acne was assessed using the MASI for 30 individuals of either sex who were receiving 20 mg of isotretinoin as a monotherapy, and were between the ages of 18 and 25. A reduction of roughly 73.4% was seen in patients who took 20 mg of oral isotretinoin for 16 weeks [56,57].

### 4.2. Topical Creams

Topical creams are the most common type of drug used to treat skin pigmentation. They are applied directly to the affected area, and can lighten or darken the skin. The main advantage of topical creams is that they can be used at home, and do not require a trip to the doctor. Additionally, they are typically less expensive than oral medications. Topical cream application, however, comes with several drawbacks. They can be messy and time-consuming to apply, and they may only sometimes be as effective as oral medications [58,59].

**Figure 7:**
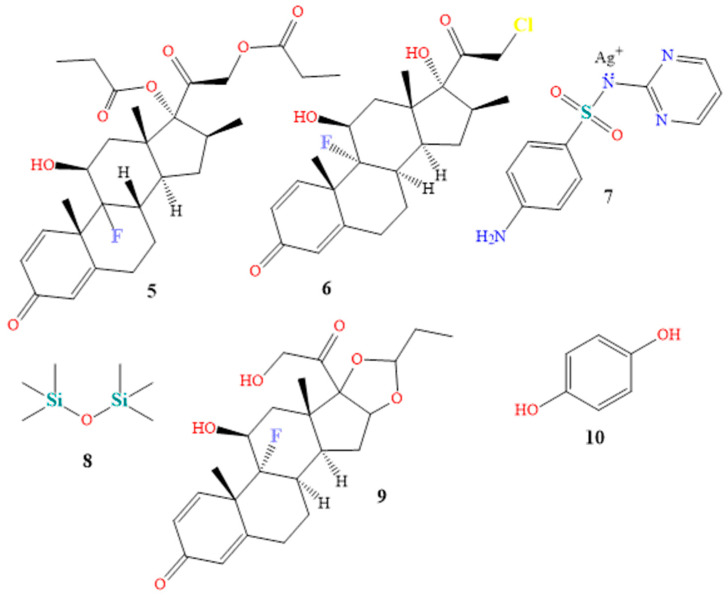
Chemical structures of betamethasone (**5**), clobetasol (**6**), silver sulfadiazine (**7**), dimethicone (**8**), triamcinolone (**9**), and hydroquinone (**10**).

Topical steroids are the most often recommended drug in dermatology. They are prescribed for various conditions, including eczema, psoriasis, atopic dermatitis, lichen simplex chronicus, intertrigo, and psoriasis, due to their immunosuppressive, anti-mitogenic, and anti-inflammatory characteristics. The dosage varies from one to three times per day. Betamethasone 0.05% (Betnovate-N^®^, chemical **5** in Figure 7) and clobetasol 0.05% (Dermovate^®^, compound **6** in Figure 7) are examples of topical steroids. The NF-Kappa B inhibitors betamethasone and clobetasol are glucocorticoids that prevent neutrophil apoptosis and demarginating. Betamethasone and clobetasol are phospholipase A2 inhibitors, which also reduce the production of arachidonic acid derivatives. Additionally, glucocorticoids encourage the anti-inflammatory gene interleukin-10 [60,61,62], a common ingredient in cream or ointment treatments. Numerous local and systemic adverse effects of topical steroids have been attributed to their continuous use [63,64,65].

A total of 15 vitiligo patients of both sexes (F:M 1.14:1) utilized betamethasone cream 0.05% twice daily throughout a three-month study. Based on a patient’s degree of minimal pigmentation/no reaction, moderate, noticeable pigmentation, or outstanding pigmentation, the improvement of each patient was rated as (25%), (25–50%), (50–75%), or (>75%), respectively. Compared to 40.0% of patients with limited pigmentation or no reaction, 46.7% and 13.3% showed a moderate or outstanding pigmentation response after therapy [66,67].

A total of 731 patients with moderate to severe plaque psoriasis, with 3% to 20% body surface area, participated in the 4-week Clobetasol Spray trial, which used two doses of clobetasol propionate spray 0.05% twice daily as treatment. The change in target plaque severity was the primary outcome measure. According to the major outcome measures scale, 80.0% of the patients in the therapy group were clear or nearly unambiguous, and showed a decrease in severity from the beginning [68,69].

A topical anti-infective cream, silver sulfadiazine (Silvadene^®^, chemical **7** in Figure 7) is primarily used to prevent and cure burn injuries. Silver sulfadiazine solution with 1% API dissolves in water. Proteins become denaturized and enzyme activity is reduced by silver ions. Additionally, silver ions bind to proteins and surface membranes, leading to membrane proton leakage and cell death. Sulfadiazine competitively inhibits PABA, a naturally occurring bacterial substance that acts as a substrate for the dihydropteroate synthase enzyme. These organisms must carry out the blocked process to produce folic acid [70]. Silver sulfadiazine exhibits broad-spectrum action against both gram-positive and gram-negative pathogens. It has been demonstrated that it promotes wound and injury repair, and has anti-infective qualities [71,72]. Twenty-seven individuals with 2° burn injuries were randomly assigned to receive silver sulfadiazine throughout a 4-week study. After four weeks of treatment, the healing condition of 2° deep dermal burn wounds were determined to be (0–25%), (26–50%), (51–75%), or (76–100%), respectively, as poor healing, moderate healing, fast healing, or excellent healing. While eight and thirteen patients showed a mild and quick recovery, respectively, six patients with 2° deep dermal burn lesions showed poor healing [73]. Mixtures of creams, shampoos, powders, mouthwash, and gels contain both an anti-infective and a steroid component, to treat skin or scalp infections [74]. Triamcinolone and dimethicone are ingredients in the drug TriHeal80^®^ (see components **8** and **9** in Figure 7). Topical corticosteroids produce similar antipruritic, anti-inflammatory, and vasoconstrictive effects [75].

Triamcinolone is a phospholipase A2 inhibitor that acts on cell membranes, to prevent the lysosomal membranes of leukocytes from rupturing. This prevents the production of arachidonic acid, which in turn lowers lipoxygenase and cyclooxygenase, while inhibiting the production of prostaglandins and leukotrienes [76,77]. Dimethicone, a silicone oil, exhibits viscoelastic qualities. It has moisturizing properties, and is utilized as a surfactant, antifoaming agent, and lubricant to cure skin irritation. To reduce the rate of water evaporation, dimethicone is used topically [78,79]. When used four times per day for two months, and monitored for another two months, triamcinolone 0.1% mouthwash successfully treated oral lichen planus in 20 patients. All effectiveness endpoints, assessed using the visual analog scale, the verbal health impact profile score, and the objective clinical score, revealed a significant improvement in the patients [80,81,82].

**Figure 8:**
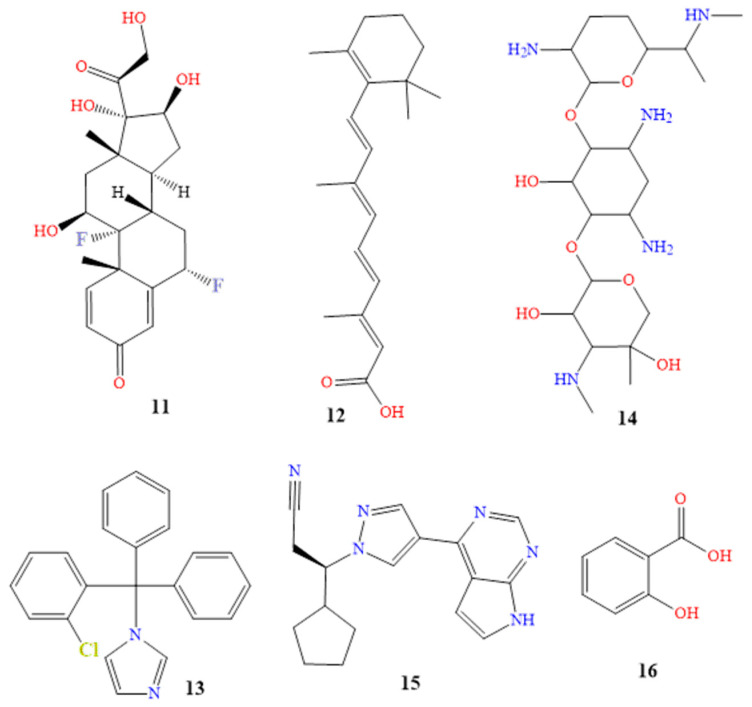
Chemical structures of fluocinolone (**11**), tretinoin (**12**), clotrimazole (**13**), gentamicin (**14**), ruxolitinib (**15**) and salicylic acid (**16**).

Under the brand name Tri-Luma^®^, a triple combination cream is sold that includes the active components tretinoin, hydroquinone, and fluocinolone in concentrations of 0.01%, 4%, and 0.05% [83]. Hydroquinone is the most frquently used skin-lightening or depigmenting substance (compound **10** in Figure 7). It treats dyschromic skin diseases such as melasma, chloasma, freckles, and post-inflammatory hyperpigmentation, by suppressing melanin production. It stops tyrosinase from converting L-3,4-dihydroxyphenylalanine into melanin, due to its structural similarity to a specific analog of melanin [83,84,85]. Fluocinolone (molecule **11** in Figure 8) treats symptoms, including itchiness, swelling, and redness caused by skin problems [86,87]. Retinol (compound **12** in Figure 8) cures skin aging. It has been shown that it might be beneficial for concerns related to skin aging. The most remarkable feature is that treatment results manifest eight to twelve months after using the tretinoin 0.1% cream preparation. The most frequent side effects of tretinoin include very slight skin irritability, and a transient, mild, and clinically uncomfortable burning sensation [85,88,89]. Sixty patients with moderate (grade 2) or severe (grade 3) melasma received treatment for eight weeks with the triple combination cream. At weeks 4, 6, and 8, the triple combination cream significantly improved the overall results, with an improvement rate of 73% (44/60). The percentage of participants who thought the triple combination cream was “excellent” as a treatment was 50%, while the most frequently mentioned adverse effects were erythema, burning, and desquamation [90].

Additionally, a 12-week open-label trial was created, to gauge the effectiveness and safety of applying topical retinol 0.15% twice daily. At the fourth, eighth, and twelve weeks, it was found that 39%, 77%, and 77% of patients, respectively, showed significant improvement. When using topical retinol, dryness, erythema, peeling, stinging, and burning were some side effects that were reported [91].

Dermatitis, eczema, rashes, and allergies are just a few of the skin conditions that TriDerm^®^ is used to treat. The swelling, redness, and itching that are brought on by these various disorders are reduced by triamcinolone. It includes corticosteroids that range in strength from mild to potent. The mechanism of action of TriDerm is composed of betamethasone, clotrimazole, and gentamicin (compounds **13** and **14** in Figure 8). This results in the antipruritic, anti-inflammatory, and vasoconstrictive effects of betamethasone, as well as the broad-spectrum bactericidal antibiotic effect of gentamicin, and the broad-spectrum antifungal effect of clotrimazole. The contents of the cell leak out when clotrimazole reacts with the fungal cell membrane. Gentamicin is an effective topical skin therapy for bacterial infections [91,92,93,94,95]. A study included 68 patients with itchy dermatoses, including atopic dermatitis, contact dermatitis, and true eczema. Of the patients, 33 received a twice-daily application of a topical cream containing betamethasone, clotrimazole, and gentamicin on the affected body parts. The effectiveness of the therapy was assessed after 7, 14, and 28 days. On the seventh day of treatment, there was a reduction in the inflammatory process and subjective symptoms. Of the 33 patients, 5 saw a scientific recovery on the fourteenth day of receiving treatment. After 28 days of therapy, the patients had fully recovered medically [96].

The main component of the topical anti-cancer drug Opzelura^®^ is ruxolitinib (compound **15** in Figure 8). A class of drugs known as Janus kinase inhibitors, which includes roxolitinib, has an effect on the immune system. JAK inhibitors may reduce the immune system’s ability to fight off infections [97,98,99,100]. JAKs serve a variety of purposes. JAK1 and JAK3 increase lymphocyte existence and differentiation, whilst JAK2 increases the signal transduction of thrombopoietin and erythropoietin. JAKs are located in the cytoplasmic region of cytokine and growth factor receptors. JAKs are also activated, and undergo cross- and tyrosine phosphorylation. Ruxolitinib has a low affinity for JAK3, but is a solid and selective inhibitor of JAK2 and JAK1. Ruxolitinib reduces the plasma levels of pro-inflammatory cytokines, and inhibits myeloproliferative neoplasms by downregulating the JAK-STAT pathway [101,102].

Randomized controlled trials recommended using ruxolitinib 1.5% cream for treating vitiligo twice daily in various patients. This was shown to demonstrate clinically excellent re-pigmentation of all body areas, including the acral region, after 24 weeks, with continued improvement through week 52. It was tolerated well in patients with long-standing high contamination [103,104].

**Figure 9:**
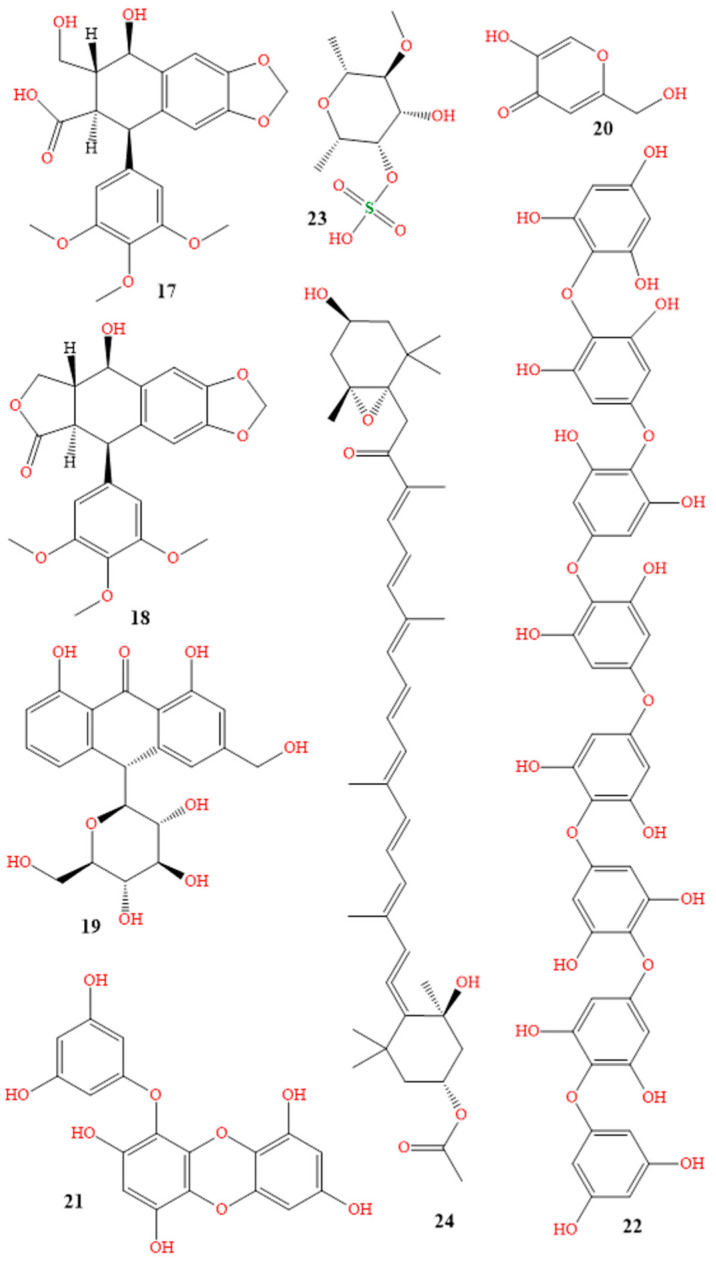
Structures of podophyllum resin (**17**), podofilox (**18**), aloin (**19**), kojic acid (**20**) phlorotannins (**21**), octaphlorethol A (**22**), fucoidan (**23**), and fucoxanthin (**24**).

Salicylic acid (Salvax^®^, compound **16** in Figure 8), podophyllum resin (Podocon-25^®^, compound **17** in Figure 9), and podofilox (Condylox^®^, compound **18** in Figure 9) are a few examples of topical keratolytics that are administered topically to the skin, to soften keratin. This facilitates the peeling of skin cells, supports the skin’s capacity to retain moisture, and aids in the treatment of dry skin conditions, and is generally used to treat skin diseases, such as psoriasis, warts, keratoses, and acne [105,106]. More topical brand names used for reducing skin pigmentation are included in Table 1.

Because of its keratolytic qualities, salicylic acid, a lipophilic B-hydroxy acid, is frequently used in cosmetic product formulations as a skin scaler for lightening. Arachidonic acid is reduced from converted prostaglandins and thromboxanes by COX-1 and COX-2 inhibitors. Salicylic acid also has anti-inflammatory and antibacterial effects [107,108]. Twenty Latin American women over the age of 18 with moderate to severe bilateral melasma participated in a small, potential randomized controlled trial to compare the efficacy of salicylic acid 20–30% scaler every two weeks, followed by up to eight weeks, in combination with hydroquinone 4% twice daily for 14 weeks, versus hydroquinone 4% alone. A narrowband reflectance spectrophotometer (Mexameter MX-16) was used to quantify the degree of pigmentation on the affected and unaffected skin on each face. The Melasma Area and Severity Index (MASI) was used to assess the severity of the melasma. Of the patients, 33% were regarded as showing a mild development and slight improvement, with 44% showing more significant progress on the peeled side. One patient (6%) was noted to show only slightly more growth on the unpeeled side. The peeled side had advanced more than the unpeeled side, according to 83% of the nonblinded patients (four somewhat, seven moderately, and four significantly). One patient (6%) thought the unpeeled side was more advanced, whereas twelve percent (12%) believed there was no difference. Table 2 shows a summary of the drugs and natural hyperpigmentation meta-analysis studies [109,110].

## 5. Natural Hyperpigmentation Treatment

Despite the wide range of therapies available, a growing number of people are choosing plants and natural items as alternatives. Plant-based and natural remedies have long been used to treat skin issues, and they are gaining popularity as a secure and efficient method to treat skin hyperpigmentation [87,116,117,118,119].

Vitamins A, B, C, and E can all be used to address skin pigmentation problems, and are necessary for healthy skin. Each vitamin, which can be obtained from foods or supplements, has specific advantages [120,121].

Niacin, pantothenic acid, and biotin are the B vitamins most frequently found in skincare products. Niacin, also known as niacinamide, is a vitamin that is used in face creams and masks, to minimize the appearance of enlarged pores, fine lines, and dullness. Pantothenic acid is also applied to dry, flaky skin, as a moisturizer. Numerous hair, nail, and skincare products include biotin [122,123]. Ascorbic acid (vitamin C), an antioxidant, inhibits tyrosinase by binding to copper, and suppressing the oxidative polymerization of melanin precursors, which prevents melanin synthesis in the melanogenesis pathway [124,125]. A statistically significant decrease from the baseline to week 16 was observed in a trial on 39 patients, using 25% L-ascorbic acid dissolved in Nmethyl-2-pyrrolidone and dimethyl isosorbide, as indicated by MASI values and mexameter data [111,112]. A particular type of vitamin E is alpha-tocopheryl acetate. When fat is subjected to oxidation, and during the spread of free radical reactions, vitamin E, a powerful chain-breaking antioxidant, prevents the synthesis of reactive oxygen species molecules [126,127,128].

*Artocarpus lakoocha* and *Glycyrrhiza glabra* extracts have been reported to exhibit tyrosinase inhibitory effects and melanin pigment reduction. For the treatment of hyperpigmentation, the combination of 9:1 *Artocarpus lakoocha* and *Glycyrrhiza glabra* decreased melanin pigment by up to 53% in B16 cells, by lowering the production of tyrosinase (TYR), microphthalmia-associated transcription factor (MITF), and tyrosinase-related protein-2 (TRP-2) [129,130,131].

Antioxidants and fatty acids included in oils such as rosehip, jojoba, and argan oil aid in reducing inflammation, and brightening the skin. Natural oils can also shield the skin from the effects of the environment, preventing further discoloration. Aloe vera also includes aloin (compound **19** in Figure 9), which has been demonstrated to lighten skin, and function well as a nontoxic hyperpigmentation therapy. Sharique described aloe vera as a natural depigmenting ingredient [132,133,134].

When used as an emollient, jojoba oil exhibits first-rate lubricity, without having an oily or greasy texture, in single-segment and emulsion structures. It can also contribute to the skin’s effective water regulation during transpiration, reducing evaporation without obstructing the passage of gases or water vapor [135,136,137,138]. According to a study, jojoba oil (or its ozonized or hydrogenated derivatives) has emollient properties. The study discovered that a significant increase in skin surface flexibility developed within 5 min and persisted for hours, suggesting a potential application in solutions for dry skin [139]. Jojoba liquid wax was found to be just as effective at treating diaper rash as triamcinolone acetonide, nystatin, neomycin, and gramicidin. Jojoba oil is also an anti-inflammatory. Due to the absence of systemic adverse effects, jojoba has the benefit of being safer [140]. Additionally, it has anti-acne and anti-psoriasis qualities, which allow the dissolution of sebum deposits through the hair follicles, due to its capacity to infiltrate the follicles, eliminate the comedones, and clear the skin [141].

In a research study, ten women used argan oil as a bandage on their skin for 28 days. None of the women experienced itching, or noticed any skin irritation or redness, demonstrating the oil’s efficacy in reducing the amount of pigmentation. These women did observe a minor decrease in melanin content in the vicinity of the bandage, though, which lends credence to the idea that the oil lessens pigmentation [113,114,115]. Licorice root extract, turmeric extract, and green tea extract are other herbal extracts high in antioxidants that help to reduce inflammation and brighten the skin. 

Since ancient times, licorice root extract has been utilized for its medical benefits, particularly for skin care. It contains glycyrrhizin, which has been shown to have antioxidant and anti-inflammatory properties [142]. Given that it is thought to help enhance skin appearance and treat some skin disorders, these qualities make it a popular ingredient in skincare products [143]. Several research studies have been conducted to determine whether licorice root extract is effective for treating skin conditions. According to a study, licorice root extract is useful for reducing hyperpigmentation and lightening the skin [144]. Atopic dermatitis symptoms may be lessened by licorice root extract, according to a different study [145]. James M. Spencer also demonstrated in his research that licorice root extract was efficient in lessening the severity of rosacea, melasma, and acne [146]. Additionally, licorice root extract reduced the appearance of black spots and redness, as was discovered in a 2019 study by Maria Yusuf Dhariwala [147].

Since ancient times, turmeric extract has been valued for its therapeutic benefits. It has a yellow tint, and various health advantages, due to the presence of the active component curcumin, when it comes to pigmentation and skin conditions. The strong anti-inflammatory qualities found in curcumin can help lessen the skin inflammation brought on by a variety of skin conditions, including psoriasis and eczema [148,149,150,151]. Antioxidants included in turmeric extract reduce oxidative damage that can cause skin aging, and pigmentation disorders such as melasma, by neutralizing free radicals [152]. Curcumin also has skin-lightening qualities. By preventing the formation of the melanin-producing enzyme tyrosinase, it can lessen hyperpigmentation, and make the skin lighter [153]. Curcuminoids, which are found in turmeric, have exfoliating qualities that aid in gently removing dead skin cells, and encourage skin regeneration, minimizing the appearance of hyperpigmentation and dark patches [154]. The use of turmeric extract to treat skin issues was the subject of a 2018 study by Alexandra R. Vaughn. In psoriasis, eczema, and acne patients, the study found that turmeric extract was beneficial in lowering skin inflammation, and enhancing skin health [155]. According to a 2018 study by Penelope J. Kallis [156], a topical cream with turmeric extract proved successful in lowering the severity of acne in patients after four weeks of treatment. 

Another organic component that has been investigated for its therapeutic advantages for the skin is green tea extract. It has many polyphenols and antioxidants, as well as anti-inflammatory and skin-protective qualities [157,158,159]. Green tea extract works in a variety of ways, to treat pigmentation issues and skin problems. Catechins and epigallocatechin gallate (EGCG), two antioxidants found in green tea, work to combat free radicals that can damage skin and speed up the aging process [160]. Green tea extract also has strong anti-inflammatory qualities that can help lessen the skin irritation brought on by a variety of skin diseases, such acne, eczema, and rosacea [161]. The EGCG in green tea extract can help inhibit tyrosinase activity, reducing the production of melanin, and thus lightening the skin [162]. Furthermore, green tea extract has been shown to offer some protection against UV radiation, which can cause skin damage and contribute to pigmentation disorders [163]. A clinical study was conducted on 11 patients to investigate the use of green tea extract in treating acne; this study found that green tea extract was effective in reducing the number of acne lesions and improving overall skin health [164]. Another study, published in 2018, found that green tea extract effectively reduced the appearance of fine lines and wrinkles in the skin [165].

Kojic acid (Enshine^®^ cream 2%, compound **20** in Figure 9) has been found to be effective in treating various skin disorders and pigmentation issues, due to its mechanism of action. It works by inhibiting the activity of tyrosinase, which reduces the production of melanin, which can help to fade dark spots and hyperpigmentation [166,167,168]. In addition to its tyrosinase-inhibiting properties, kojic acid has antioxidant and anti-inflammatory properties; these can be particularly beneficial to individuals with acne, rosacea, and other inflammatory skin conditions [169,170,171,172]. One study, published in 2016 by Peter J. Gust, evaluated the efficacy of a cream containing 2% kojic acid, 10% glycolic acid, and 2% hydroquinone for treating melasma. The study involved 40 participants, who applied the cream twice daily for 12 weeks. The results showed a significant reduction in the severity of melasma in the treated group, compared to the control group, with no reported adverse effects [173]. In another study, Tamara Searle investigated the use of a cream containing 2% kojic acid, 1% arbutin, and 5% vitamin C to treat age spots. The study involved 60 participants, who applied the cream twice daily for 12 weeks. The results showed a significant reduction in the number and severity of age spots in the treated group, compared to the control group, with no reported adverse effects [174]. Several herbs and naturally occurring substances that are commonly used in skincare products, for their ability to lighten skin and reduce hyperpigmentation, are listed in Table 3.

Phlorotannins (compound **21** in Figure 9) from brown algae (brown seaweed) play a crucial role in the reduction of hyperpigmented effects, and the prevention of premature skin aging. They protect the skin from the sun’s infrared and blue rays.

Additionally, they promote cellular energy generation, which raises the oxygenation of the skin. Through this technique, the skin’s overall appearance and cell innovation are improved. Their antioxidant action prevents the collagen that firms the skin from degenerating [175,176,177]. Phlorotannins have been studied for their impact on skin conditions and pigmentation, in clinical trials and meta-analyses. A randomized, double-blind, placebo-controlled study in 2022 discovered that women with dry skin saw improvements in skin hydration, suppleness, and wrinkle formation with an Ecklonia cava (Phaeophyceae) extract high in phlorotannins. Another randomized, double-blind, placebo-controlled study discovered that a phlorotannin-rich Ascophyllum nodosum extract reduced face pigmentation, and enhanced skin suppleness, in women with age spots [178,179].

Researchers have looked into the potential for marine-derived compounds from Undaria pinnatifida, Octopus vulgaris, and Sargassum polycystum, to improve skin pigmentation, as well as to possess antioxidant, anti-inflammatory, and immunomodulatory capabilities. These substances (compounds **22**–**24** in Figure 9) also contain octaphlorethol A, fucoidan, and fucoxanthin [180,181,182,183,184,185]. Numerous studies have looked into how octaphlor-ethol A affects skin conditions and pigmentation. According to one study, in human melanoma cells, octaphlorethol A inhibited the formation of melanin, and decreased skin pigmentation. In a different study, mice with atopic dermatitis showed enhanced skin barrier function and reduced inflammation when treated with octaphlorethol A [179,186]. Additionally, a cream containing fucoidan and marine collagen enhanced skin hydration, suppleness, and wrinkle formation in women with dry skin, according to a randomized, double-blind, placebo-controlled research study [187]. Additionally, a cream containing fucoidan and marine collagen enhanced skin hydration, suppleness, and wrinkle formation in women with dry skin, according to a randomized, double-blind, placebo-controlled research study [188]. Fucoxanthin, a carotenoid pigment, was found to be associated with a significant decrease in the severity of melasma in a review of 11 randomized controlled studies. To corroborate these findings, more research is required, as the authors stated that the quality of the inclusive studies was generally low [189,190,191].

**Table 3:** Plants discovered to treat hyperpigmentation in the past ten years.

Name of Plant	Family	Growth Place	Active Compounds	Type of Pigmentation Targeted
*Angelica sinensis* [192]	Apiaceae	East Asia	4-ethylresorcinol, 4-ethylphenol, 1-tetradecalnol	Hyperpigmentation agent combating skin-darkening. Study on Melan-A cells
*Artocarpus* [193]	Moraceae	Southeast Asia	Artocarpin, cudraflavone C, artocarpanone	TYR inhibitor. Hyperpigmentation–skin-whitening agents
*Callicarpa longissima* [194]	Lamiaceae	Southeast Asia	Carnosol	Antimelanogenesis in B16F10. Hyperpigmentation agents
*Crataegus azarolus* [195]	Rosaceae	European	Ursolic acid, hyperoside, virtexin-2″-O-rhamnoside	Antimelanogenesis in B16F10. Hyperpigmentation agents
*Cyperus rotundus* [196]	Cyperaceae	Africa, France, Austria, southern Asia	Valencene, camphene, carryophyllene oxide	Antimelanogenesis mechanism via the ion-channels in B16F10. Hyperpigmentation agents
*Juniperus chinensis* [197]	Cupressaceae	China, Myanmar, Russian, Korea	Widdrol	α-Melanocyte-stimulating hormone inhibition in B16F10 and TYR. Hyperpigmentation agents
*Morus nigra* [198]	Moraceae	Iberian Peninsula	Isoquercitrin, rutin, chlorogenic acid	Inhibit mushroom TYR. Hyperpigmentation agents
*Oryza sativa* [199]	Poaceae	China	p-Coumaric, ferulic	Antimelanogenesis in B16F10 melanoma by TYR. Hyperpigmentation agents
*Passiflora edulis* [200]	Passifloraceae	Brazil, Paraguay, Argentina	Piceatannol, resveratrol, quercetin	Antimelanogenesis in melanoma cells. Hyperpigmentation agents
*Salvia officinalis* [201]	Lamiaceae	Mediterranean region	7a-methoxyrosmanol, isorosmanol	Antimelanogenesis in B16. Hyperpigmentation agents
*Sesamum indicum* [202]	Pedaliaceae	Africa, India	Sesamol	Antimelanogenesis in B16F10. Hyperpigmentation agents
*Punica granatum* [203]	Lythraceae	Mediterranean	Punicalgin	Antimelanogenesis in Melan-A. Hyperpigmentation agents
*Litchi chinensis* [204]	Sapindaceae	China, India, Bangladesh, Vietnam, Thailand, Malaysia, Indonesia, Pakistan, Cambodia, Bangladesh, Himalayas	Rosmarinc acid, gallic acid	Suppressed melanin production in B16F10 melanoma cells. Hyperpigmentation agents

## 6. Modern Skin Pigmentation Treatments and Promising New Technologies

The preferred method of treatment for problems with skin pigmentation has long been laser therapy. The melanin in the skin can be reduced and evened out by lasering the afflicted area, leading to a more even complexion. As time goes on, technological developments mean that lasers are more and more efficient. Today, pigmentation can be targeted in deeper, more covert locations, thanks to laser technology. For instance, lasers can now be used to target pigment under the skin’s surface, without causing irritation or damage to the skin! As a result, problems such as age spots and sun damage can be treated without any negative consequences or discomfort. To remove obstinate pigmentation, the most recent lasers combine optical energy with intense pulsed light (IPL). This is an exciting development, as it makes it possible to treat patients more quickly and effectively than ever before [205,206,207].

Topical creams and serums are among the newest and most promising therapies for skin pigmentation. These remedies include substances such as niacinamide, kojic acid, licorice extract, and mulberry extract that are especially made to fight pigmentation. These cutting-edge chemicals have the potential to significantly reduce dark spots, lighten skin tone, and enhance the skin’s overall clarity and texture. For instance, kojic acid can limit tyrosinase activity, which helps to prevent hyperpigmentation from occurring, and niacinamide can suppress melanin formation, which helps to reduce skin discoloration. In order to prevent the skin from experiencing any unpleasant reactions or side effects, the treatment should also be free of parabens and other harmful preservatives [208,209,210]. In order to diminish pigmentation, micro-needling is a process used to increase the skin’s natural collagen and elastin production. In order to provide a more exact therapy, the technique now involves using specialized instruments. Small needles are used in the procedure, to puncture the skin and create microscopic channels that can only be seen under a microscope. This straightforward procedure enhances collagen synthesis, while promoting the skin’s natural ability to mend itself. Overall, with excellent outcomes, micro-needling is quickly rising to the top of the list of popular methods for lightening skin [211,212,213]. Typically, chemical peels are used to remove the top layers of skin, which lessens the visibility of dark patches. Combination treatments, however, are far more effective for the skin. Combination therapies are proving to be even more effective at minimizing dark spots. These combination treatments include several acids, such as glycolic acid and lactic acid, which, when used together, can be much more potent than when used separately. These combined therapies, which neither lasers nor light-based devices can currently offer, can help with both facial discoloration, and uneven pigmentation on other parts of the body, with only one treatment [214,215].

With the development of new technology, the future of skin pigmentation therapies appears to be more promising than ever. One of the most promising new therapies for diseases of skin pigmentation is plasma pen therapy. Freckles, age spots, sunspots, and melasma can all be treated using this technique, which removes pigment from the skin by means of a targeted plasma energy beam. Compared to previous therapies, this one is less intrusive, and has fewer adverse effects [216,217,218].

The use of radiofrequency therapies to treat diseases of skin pigmentation is growing in popularity. The appearance of dark areas, and the overall tone and texture of the skin, can be improved by this technology, which uses radio waves to break down melanin deposits in the skin. Radiofrequency treatments are quick, non-invasive, safe, and do not entail a long recovery time [218,219].

## 7. Conclusions

Skin pigmentation is the phrase used to describe the color of someone’s skin, which is determined by how much melanin is produced by melanocytes in their skin. The two main types of melanin are eumelanin and pheomelanin. Eumelanin, which defends against skin cancer and sun damage, is the cause of dark skin tones. Pheomelanin, on the other hand, causes lighter skin tones, has the ability to regulate body temperature, and provides protection against skin cancer. The causes of skin pigmentation include genes, sun exposure, hormonal changes, skin traumas, and some medications. Other anomalies of skin pigmentation, such as vitiligo, albinism, and melasma, are brought on by genetic changes. Hyperpigmentation and hypopigmentation are the two basic types of skin pigmentation. When melanin is produced excessively, it causes hyperpigmentation, which manifests as darker spots of skin. This can be influenced by the sun, hormonal changes, and particular medications. Hypopigmentation, which results in lighter regions of skin, is brought on by melanin loss. This can be caused by genetic conditions, skin traumas, and certain medicines. In recent years, we have seen an increase in the use of oral tablets and topical lotions to treat skin pigmentation. In addition to being more costly, oral drugs have more serious adverse effects than topical treatments. Clinical studies and meta-analyses have demonstrated that oral medication in the form of tablets containing tranexamic acid and isotretinoin can be used to treat a variety of skin disorders, such as eczema, melasma, and other associated conditions. Because they are often less expensive than oral drugs, and can be used at home, topical creams are the most popular type of medication used to treat skin pigmentation. The most frequently prescribed treatments for skin diseases are topical steroids, which have a number of disadvantages compared to oral prescriptions, including messiness, and an occasionally lesser efficacy. Additionally, caution must be exercised, to prevent the negative consequences of the continuous usage of topical steroids. Additionally, clinical studies have shown that the topical versions of salicylic acid, tretinoin, betamethasone, clobetasol, triamcinolone, dimethicone, fluocinolone, hydroquinone, and silver sulfadiazine are useful in treating skin conditions. Along with octaphlorethol A, fucoidan, and fucoxanthin marine extracts, natural extracts such as rosehip, jojoba, argan oil, aloe vera, licorice root, curcumin, green tea, and kojic acid have potent anti-inflammatory properties that can help reduce inflammation in the skin caused by a variety of skin conditions, such as acne, eczema, and rosacea. With the development of technology, laser therapy has long been the preferred method of treating skin pigmentation problems. Lasers are now more efficient, thanks to ongoing technical developments that provide higher precision, faster healing times, and better outcomes. While micro-needling is a process intended to promote the skin’s natural synthesis of collagen and elastin, to diminish pigmentation, topical creams and serums contain cutting-edge chemicals created to combat pigmentation.

Combination treatments, which use a combination of acids to lessen the appearance of dark spots, are becoming more and more popular for treating skin pigmentation disorders. Promising technologies such as plasma pen therapy and radiofrequency treatments are also gaining popularity.

## Figures and Tables

**Table 1:** Topical brands of drug for the treatment of skin pigmentation.

Class	Generic Name	Brand Names^®^	Dosage Form
Topical steroids	Betamethasone	Etnovate, Diprolene, Luxiq, Beta-Val, Diprolene AF	Cream, gel, ointment, lotion
	Clobetasol	Dermovate, Clobex, Olux, Olux-E, Temovate, Clobevate, Clodan, Cormax, Cormax Scalp, Embeline, Embeline E, Impeklo, Tovet	Solution, spray, ointment, gel, foam, lotion, cream, shampoo
	Triamcinolone acetonide	DermasilkRx SDS Pak, Dermasorb TA, DermaWerx SDS Pak, Kenalog, Oralone, Trianex, Triderm	Cream, ointment
Topical anti-infectives	Silver topical	SilvaSorb, Aceso Ag, Solox	Cream, gel, foam
Topical steroids with anti-infectives	Dimethicone and triamcinolone topical	Yaliira Pak, Ellzia Pak, TriaDime-80, TriHeal-80	Creams, shampoos, powders, gels
Topical depigmenting agents	Fluocinolone, hydroquinone and tretinoin topical	Tri-Luma, Triderma	Cream
	Hydroquinone topical	Melquin HP, Alera, EpiQuin Micro, Esoterica, Hydro-Q, Melamin, Melpaque HP, Nuquin HP, AMBI Fade, Blanche, Esoterica Nighttime, Glytone, Lustra-Ultra, Melamin-C, NeoStrata HQ Skin Lightening, Olivia Quido, Fade cream, Remergent HQ	Cream
Topical keratolytics	Salicylic acid topical	Bensal HP, KeralytGel, Salex, Acnex, Aliclen, DHS Salicylic Acid 3%, Durasal, Keralyt Shampoo, Stri-Dex, Akurza, DermalZone, Dr Scholl’s, Fostex, Freezeone, Rayasal, Salvax, Stridex,	Liquid, soap, cream, lotion, foam
	Podophyllum resin topical	Podocon-25, Podofin, Pododerm	Topical solution
	Podofilox topical	Condylox	Topical gel, topical solution

**Table 2:** Summary of drug and natural hyperpigmentation meta-analysis studies.

Drug	Method of Evaluation	Duration	Performed Organism	Activity	Skin Disorder	Ref.
Tranexamic acid orally	Clinical and photographic	6 months	Human	10.8% excellent, 54% good, and 31.1% fair	Melasma	[41,42]
Topical tranexamic acid	MASI and chromameter	12 weeks	Human	Improvement in the fourth and eighth weeks	Melasma	[45,46,47]
Isotretinoin orally	MASI	16 weeks	Human	Reduction of roughly 73.4%	Acne	[56,57]
Betamethasone cream	Clinical	3 month	Human	40.0% no reaction, 46.7% moderate and 13.3% severe response	Vitiligo	[66,67]
Clobetasol propionate spray	Clinical	4 weeks	Human	80.0% of the patients in the therapy group had a decrease in severity from the beginning	Plaque psoriasis	[68,69]
Silver sulfadiazine cream	Clinical	4 weeks	Human	30% mild recovery, 48% quick recovery, 22% poor healing	2° burn injuries	[75]
Triamcinolone mouthwash	Visual analog scale	2 months	Human	Significant improvement in all patients	Oral lichen planus	[80,81,82]
Topical tretinoin, hydroquinone, and fluocinolone	Clinical	8 weeks	Human	Improvement rate of 73% at weeks 4, 6, and 8	Melasma	[90]
Tretinoin topically	Clinical	12 week	Human	77% of patients showed significant improvement	Melasma	[91]
Topically betamethasone, clotrimazole, and gentamicin	Clinical	28 days	Human	At 7th day, a reduction in inflammation; at 14th day, scientific recovery in 42% of patients; at 28th day, the patients had fully recovered medically	Itchy dermatoses, including atopic dermatitis, contact dermatitis, and true eczema	[96]
Ruxolitinib	Clinical randomized controlled trials	52 week	Human	Clinically excellent repigmentation after 24 weeks, with continued improvement through week 52	Vitiligo	[103,104]
Salicylic acid	Mexameter and MASI	14 weeks	Human	83% believed that the peeled side had advanced more than the unpeeled side; 6% thought the unpeeled aspect was more advanced; 11% believed there was no difference	Bilateral melasma	[107,108]
L-ascorbic acid	Mexameter and MASI	16 weeks	Human	Decrease from baseline	Enlarged pores, fine lines, and dullness	[111,112]
Argan oil	Clinical	28 days	Human	Patients observed a minor decrease in melanin content in the vicinity of the bandage	Reducing the amount of pigmentation	[113,114,115]

## Data Availability

Not applicable.
